# The Pentatricopeptide Repeat Protein OsPPR674 Regulates Rice Growth and Drought Sensitivity by Modulating RNA Editing of the Mitochondrial Transcript *ccmC*

**DOI:** 10.3390/ijms26062646

**Published:** 2025-03-14

**Authors:** Jinglei Li, Longhui Zhang, Chenyang Li, Weijun Chen, Tiankang Wang, Lvni Tan, Yingxin Qiu, Shufeng Song, Bin Li, Li Li

**Affiliations:** 1 Longping Branch, College of Biology, Hunan University, Changsha 410125, China; jingleili065@hnu.edu.cn (J.L.); greeni02@hnu.edu.cn (L.T.); qq028@hnu.edu.cn (Y.Q.); shufengsong@hhrrc.ac.cn (S.S.); 2 State Key Laboratory of Hybrid Rice, Hunan Hybrid Rice Research Center, Hunan Academy of Agricultural Sciences, Changsha 410125, China; zhanglh0623@163.com (L.Z.); 24110901000050@hainanu.edu.cn (C.L.); chenwj307@hnu.edu.cn (W.C.); wangtiankang2007@126.com (T.W.)

**Keywords:** rice (*Oryza sativa* L.), RNA editing, PPR protein, drought stress adaptation, molecular breeding

## Abstract

The P-type pentatricopeptide repeat (PPR) proteins are crucial for RNA editing and post-transcriptional regulation in plant organelles, particularly mitochondria. This study investigates the role of *OsPPR674* in rice, focusing on its function in mitochondrial RNA editing. Using CRISPR/Cas9 technology, we generated *ppr674* mutant and examined its phenotypic and molecular characteristics. The results indicate that *ppr674* exhibits reduced plant height, decreased seed-setting rate, and poor drought tolerance. Further analysis revealed that in the *ppr674* mutant, RNA editing at the 299th nucleotide position of the mitochondrial *ccmC* gene (C-to-U conversion) was abolished. REMSAs showed that GST-*PPR674* specifically binds to RNA probes targeting this *ccmC*-299 site, confirming its role in this editing process. In summary, these results suggest that *OsPPR674* plays a pivotal role in mitochondrial RNA editing, emphasizing the significance of PPR proteins in organelle function and plant development.

## 1. Introduction

Maintaining intracellular energy homeostasis is a prerequisite for cells to sustain normal physiological functions. The mitochondrion, as the primary energy-producing organelle, plays a pivotal role in plant growth and development by generating ATP through oxidative phosphorylation [1]. The biogenesis and function of mitochondria require the intricate coordination between nuclear and mitochondrial genomes to ensure proper assembly and activity of the organelle [2]. This complex process involves two major transcription systems: the nuclear-encoded RNA polymerase (NEP) and the mitochondrial-encoded RNA polymerase (MEP), which coordinate gene expression at various stages [3]. Mature mitochondrial mRNAs undergo extensive post-transcriptional modifications, including RNA editing, splicing, processing, and stabilization [4]. Among these processes, RNA editing—primarily cytidine-to-uridine (C-to-U) conversions—plays a crucial role in refining mitochondrial gene transcripts to ensure proper protein functionality. Furthermore, the mitochondrial RNA editing process is vital for plant adaptation to environmental stresses, as it influences the efficiency of the electron transport chain and ATP production, thereby affecting stress tolerance and growth [5,6,7]. Disruptions in mitochondrial RNA editing can lead to compromised energy production, resulting in growth retardation and increased sensitivity to abiotic stresses, such as drought and high temperatures. The pentatricopeptide repeat (PPR) protein family plays a key role in editing mitochondrial transcripts. These proteins bind to specific RNA sequences and guide the editing machinery to modify mitochondrial transcripts. This process is essential for maintaining mitochondrial function, energy production, and stress tolerance. Furthermore, PPR proteins also regulate the stability of mitochondrial transcripts, ensuring proper mitochondrial gene expression.

The PPR gene family in the rice genome exhibits a high degree of complexity and a rich diversity of members, providing an abundant genetic resource for rice genetic research and breeding [8]. The key structural feature of PPR proteins is a tandem array of repeat units, each consisting of approximately 35 amino acids, which are classified into P-type and PLS-type motifs. Some PPR proteins further contain expansions of the PLS motif, known as E and DYW domains. These conserved motifs are thought to have specific functions, including recognition and binding of target sequences (P-/PLS-type motifs), interaction with other proteins (E domain), and functioning as a cytidine deaminase (DYW domain) [9,10]. The E2 subgroup of PPR proteins is distinct from the E subgroup in that it specifically targets RNA-editing sites, playing a crucial role in the C-to-U editing process in plant organelles, which is essential for maintaining proper mitochondrial and chloroplast functions [8]. Mutations in these conserved regions may lead to dysfunction of PPR proteins, thereby causing defects in the RNA-editing processes of organelles [11]. Mutations in mitochondria-targeted PPR genes can significantly impact plant growth and development by disrupting mitochondrial RNA processing, leading to defects in respiratory chain function and energy metabolism. Several mitochondria-localized PPR genes, such as *OsPPR19*, *DEK48*, *DEK35*, *Emp10*, and *DWEORG1*, are essential for mitochondrial biogenesis and function. Loss-of-function mutations in some of these genes lead to severe phenotypic alterations, including impaired seed development, growth retardation, and increased stress sensitivity due to defects in mitochondrial RNA editing and splicing [12,13,14,15,16]. Additionally, the mitochondrial PPR protein PPR40 has been implicated in modulating plant responses to abiotic stress through its role in mitochondrial function and ROS homeostasis [17].

In addition to their role in mitochondrial biogenesis, several PPR genes such as *Rf2*, *Rf3*, *Rf4*,*Rf5*, and *Rf20* are involved in cytoplasmic male sterility (CMS) regulation in crops, where their mutations result in pollen sterility and defective mitochondrial transcript processing, which ultimately affects fertility restoration mechanisms [18,19,20,21,22]. Furthermore, stress-sensitive PPR genes, such as *OsNBL3*, *PPR035,* and *PPR406*, influence mitochondrial function under varying environmental conditions. These genes have been shown to be involved in the regulation of mitochondrial RNA editing, which is crucial for plant development and environmental adaptation [23,24]. Although significant progress has been made in understanding the involvement of PPR proteins in mitochondrial function, the precise regulatory mechanisms underlying their roles in mitochondrial gene expression and energy metabolism remain largely unexplored. Further investigation into the diverse functions of mitochondria-targeted PPR proteins will provide valuable insights into improving plant stress tolerance and productivity.

Phylogenetic analysis of the rice PPR gene family identified a small clade containing two closely related genes, *PPR756* and *OsPPR674*. *PPR756* is known to ensure normal rice development through RNA editing at mitochondrial gene sites. To explore *OsPPR674*, we generated CRISPR/Cas9 mutants. These mutants exhibited reduced plant height, decreased seed-setting rate, and poor drought tolerance. Mechanistic studies showed that *OsPPR674* regulates RNA editing of the mitochondrial gene *ccmC* and plays a role in cytochrome c synthesis.

## 2. Results

### 2.1. OsPPR674 Encodes a PPR Protein Belonging to the E Subfamily

**Figure 1:**
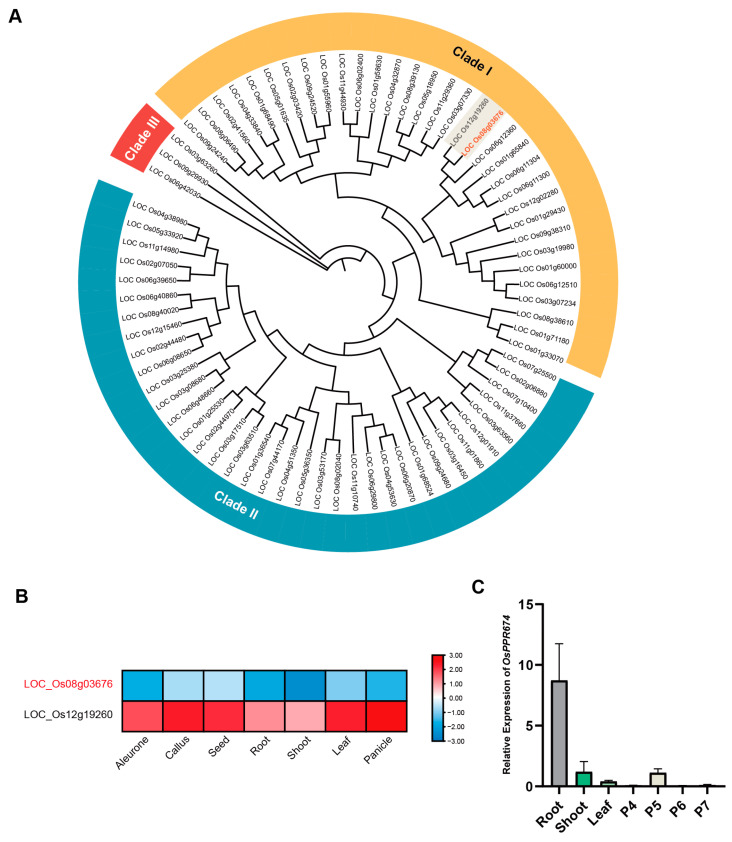
Phylogenetic and expression analysis of *OsPPR674* and its homologs: (**A**) Phylogenetic tree of E2 subfamily PPR proteins in rice. (**B**) Expression profiles of *OsPPR674* (LOC_Os08g03676) and *PPR756* (LOC_Os12g19260) in rice tissues from the rice expression database. (**C**) Reverse Transcription Quantitative Polymerase Chain Reaction (RT-qPCR) analysis of OsPPR674 expression in various tissues, including roots, stems, leaves, and panicles, at developmental stages P4–P7.

By analyzing the protein sequence of *OsPPR674*, we identified it as a member of the E2 subfamily of PPR proteins. Consequently, we constructed a phylogenetic tree for the E2 members of the rice PPR family (Figure 1A).

The tree divides the E2 members of the PPR family into three distinct clades, each with unique characteristics: Clade I contains a large number of highly conserved genes, likely involved in basic cellular processes such as RNA editing, transcription, and translation in organelles, indicating their important role in maintaining organelle genome integrity and appropriate gene expression; Clade II has fewer genes and may be involved in specific RNA metabolic processes, stress responses, and developmental functions, suggesting specialized roles in rice; Clade III contains the fewest genes, indicating highly specialized functions, possibly targeting specific RNA molecules for editing or regulation under certain conditions or in specific tissues. *OsPPR674*, located in Clade I, reflects its high conservation and important role in basic cellular processes, making it a key focus for further research into the fundamental mechanisms of RNA metabolism in rice.

We then found that in this phylogenetic tree, the protein closest in evolutionary relationship to *OsPPR674* is the previously studied *PPR756*, which affects rice growth and development, particularly pollen fertility. *PPR756* knockout plants exhibit slow growth during early vegetative development, with erect and darker green leaves, high accumulation of chlorophyll a and b, and during the reproductive stage, they show pollen sterility and low seed-setting rate. The loss of *PPR756* function eliminates RNA editing of three mitochondrial genes (*atp6*, *ccmC*, and *nad7*), and the defective C-to-U conversion leads to incorrect amino acid retention, which can cause pollen abortion [25]. Therefore, we considered whether *OsPPR674* might have similar functions to *PPR756*. We analyzed the spatial expression profiles of these two genes through expression data in the RED database and intriguingly found significant differences (Figure 1B). *PPR756* is highly expressed in reproductive organs, while *OsPPR674* is expressed at low levels throughout the entire growth period. Although these two genes share similar protein structures, differences in their expression patterns suggest that they may have divergent functional roles. To analyze the tissue-specific expression pattern of *OsPPR674*, RT-qPCR was performed using wild-type rice samples collected from various tissues, including roots, stems, leaves, and panicles at different developmental stages (P4–P7). The results indicated that *OsPPR674* was expressed at low levels in all tested tissues except for roots, where its expression was notably high, suggesting a potential role in root-specific metabolism and growth processes (Figure 1C).

This result is inconsistent with our previous predictions using the database, leading us to reconsider the function of this gene. It is possible that *OsPPR674* may affect the growth and development of rice at certain stages through high expression in the roots during specific periods.

### 2.2. Expression Pattern and Subcellular Localization of OsPPR674

**Figure 2:**
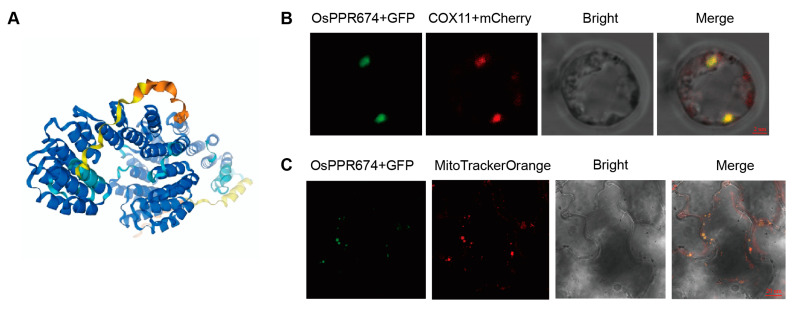
Analysis of OsPPR674 structural and expression: (**A**) Predicted protein structure of OsPPR674 using AlphaFold3. Colors from blue to orange represent confidence levels from high to low. (**B**) Subcellular localization of *OsPPR674*-GFP in rice protoplasts, co-localized with the mitochondrial marker *COX11*-mCherry (red). Bars, 2 µm. (**C**) Transient expression of *OsPPR674*-GFP in tobacco cells, co-localized with the mitochondrial marker MitoTracker Orange (red). Bars, 20 µm.

The AlphaFold3 prediction of the OsPPR674 protein structure indicates significant conservation across most regions, implying their essential role in the protein’s function. The model shows a structured arrangement of alpha helices and beta sheets, which are key to understanding OsPPR674’s activity in rice cell (Figure 2A).

To experimentally verify this prediction, a transformation vector expressing an *OsPPR674*-GFP fusion protein was constructed and transiently expressed in rice protoplasts. Fluorescence microscopy of the transformed protoplasts showed that the green fluorescence signal from *OsPPR674*-GFP perfectly co-localized with the red fluorescence emitted by *COX11*-mCherry, a validated mitochondrial expression marker (Figure 2B). This co-localization strongly supported the mitochondrial localization of OsPPR674. To further confirm this observation, the *OsPPR674*-GFP construct was transiently expressed in tobacco (*Nicotiana benthamiana*) leaves. Confocal microscopy analysis revealed that the green fluorescence of GFP overlapped with the orange fluorescence of MitoTracker Orange, a mitochondria-specific dye (Figure 2C).

Together, these results primarily demonstrate that the structure of the OsPPR674 protein is conserved and stably localized in mitochondria.

### 2.3. OsPPR674 Mutants Exhibit Reduced Plant Height and Seed-Setting Rate

**Figure 3:**
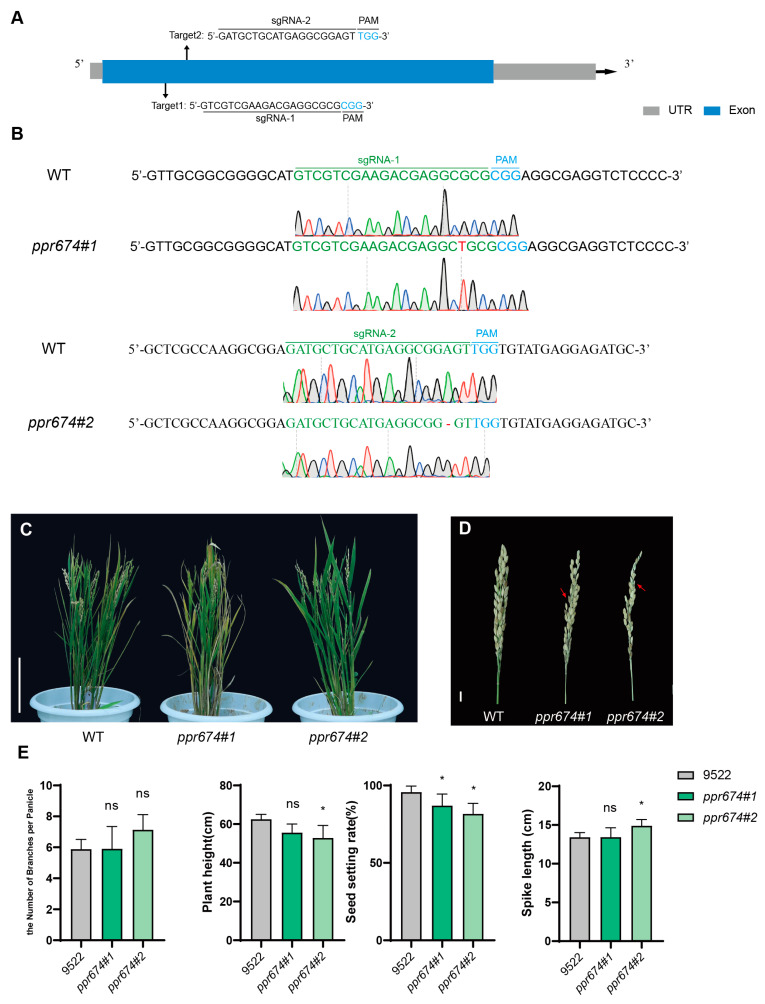
CRISPR-Cas9-mediated gene editing and phenotypic analysis of *OsPPR674* mutants: (**A**) The sequences and target sites of sgRNAs sgRNA-1 and sgRNA-2 within the *OsPPR674* gene. sgRNA sequences are indicated in black, with PAM sequences in blue. (**B**) Sequencing results of the sgRNA target sites from various mutant lines, showing both wild-type (WT) and mutated sequences. (**C**) Phenotypic characterization of whole rice plants, displaying 9522 (wild type) and *ppr674* mutants at the seedling stage. Bars, 20 cm. (**D**) Phenotypic characterization of rice panicles, showing mature panicles from 9522 (wild type) and *ppr674* mutants. Bars, 1 cm. (**E**) Comparison of key agronomic traits between 9522 and *ppr674* mutants, with data presented as means ± SD from three independent biological replicates. Significant differences are marked with asterisks (* *p* < 0.05, Student’s *t*-test).

CRISPR/Cas9 technology was employed to generate four homozygous mutant lines of *OsPPR674*, with two target sites designed in its exon regions. Sequence analysis confirmed the presence of targeted mutations in these lines (Figure 3A,B). Phenotypic analysis revealed that *OsPPR674* mutants exhibited significantly reduced plant height and seed-setting rate compared to wild-type plants. Specifically, the mutants were approximately 10–15% shorter and showed a 10–15% decrease in the seed-setting rate (*p* < 0.01) (Figure 3C–E).

The observed phenotypic defects indicate that *OsPPR674* may be associated with normal growth and reproductive development, likely due to its role in mitochondrial metabolism.

### 2.4. OsPPR674 Mutants Exhibit Drought Sensitivity

Promoter motif analysis of *OsPPR674* revealed the presence of multiple environmental response elements, including light-responsive, hormone-responsive, and drought-responsive motifs. These findings suggest that *OsPPR674* may play a role in abiotic stress responses (Supplementary Figure S1).

**Figure 4:**
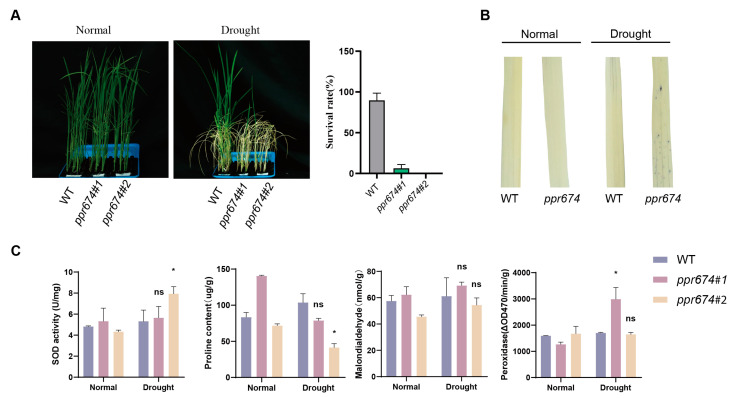
Functional analysis of *OsPPR674* under drought stress conditions: (**A**) Seedling phenotypes post-drought treatment with 20% PEG6000 for 7 days followed by 15 days of recovery. (**B**) NBT staining indicating reactive oxygen species (ROS) accumulation in 9522 (wild type) and *ppr674* mutants after 3 hours of drought treatment with 20% PEG6000. (**C**) Physiological assays measuring proline content (μg/g), superoxide dismutase (SOD) activity (U/mg), peroxidase activity (ΔOD470/min/g), and malondialdehyde (MDA) content (nmol/g) under both normal and drought conditions after 24 hours of treatment with 20% PEG6000. Statistical significance is denoted by asterisks (* *p* < 0.05); ns signifies non-significant differences.

To test this hypothesis, drought treatments were applied to wild-type and mutant plants. Under 20% PEG6000 treatment for 7 days followed by a 15-day recovery period, the survival rate of mutants was closely 0%, while wild-type plants exhibited a survival rate of approximately 80% (Figure 4A). Further analysis revealed significant physiological differences between mutants and wild-type plants under drought stress. NBT staining showed increased ROS accumulation in mutants, indicating impaired oxidative stress management (Figure 4B). Under drought conditions, the *ppr674* mutants exhibited significantly lower survival rates compared to the wild type, highlighting their heightened sensitivity to water scarcity. Quantitative RT-PCR analysis revealed that *OsPPR674* expression in wild-type plants was most significantly upregulated under drought stress compared to salinity and high-temperature conditions (Supplementary Figure S2).

To investigate the physiological responses of the *ppr674* mutants to drought stress, we measured the activities of superoxide dismutase (SOD) and peroxidase (POD), as well as the levels of malondialdehyde (MDA) and proline (Figure 4C). Physiological assays revealed increased superoxide dismutase (SOD) and peroxidase (POD) activities in the mutants. These increases in antioxidant enzyme activities are indicative of the plants’ response to oxidative stress and underscore the mutants’ heightened sensitivity to drought conditions. While malondialdehyde (MDA) levels, a marker of lipid peroxidation, were measured in both mutant lines, the trends were inconsistent relative to the wild type, with one mutant line showing an increase and the other a decrease. Additionally, the mutants showed decreased proline concentrations, a critical osmoprotectant that helps maintain osmotic balance and cellular integrity under dehydration stress. The reduced proline levels further suggest an impaired ability to regulate osmotic pressure, which is essential for preserving cell turgor and protecting cellular components from the detrimental effects of drought.

### 2.5. OsPPR674 Participates in RNA Editing of the Mitochondrial Gene ccmC

**Figure 5:**
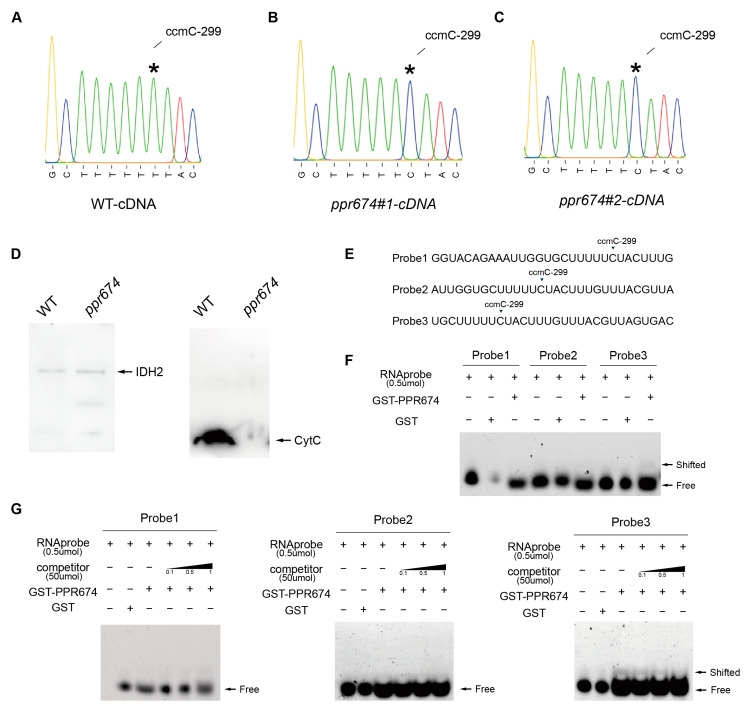
Examination of *ccmC* editing and protein interactions in *ppr674* mutants: (**A**–**C**) Sequences of the ccmC gene cDNA from WT and ppr674 mutants, with the editing site at position 299 indicated by an asterisk (*). (**D**) Western blot analysis showing protein levels of IDH2 and cytochrome c in WT and *ppr674* mutants. (**E**) FITC-labeled RNA probes targeting the *ccmC*-299 site. (**F**) REMSA demonstrating GST-PPR674 binding to the probes. (**G**) Competitive REMSA confirming binding specificity with unlabeled probes.

Given its conserved PPR domains and mitochondrial localization, OsPPR674 was hypothesized to function in mitochondrial RNA editing. To test this hypothesis, we examined the RNA-editing status of 81 mitochondrial genes in both wild-type (WT) and *OsPPR674* mutant lines. Our analysis revealed that the *OsPPR674* mutant exhibits a specific and stable loss of RNA editing at position 299 in the *ccmC* gene, while other mitochondrial editing sites remain unaffected (Supplementary Figure S3). Sequencing analysis of mitochondrial transcripts in mutants revealed the loss of C-to-T editing at position 299 in the *ccmC* cDNA, indicating the absence of C-to-U editing at the corresponding site (position 299) in the *ccmC* transcript (Figure 5A–C). This site-specific editing defect was exclusive to *OsPPR674* mutants, suggesting a direct role of the protein in RNA editing. *ccmC* encodes a cytochrome c oxidase subunit in mitochondrial complex III, which is essential for the mitochondrial electron transport chain [26]. The absence of RNA editing at *ccmC*-299 likely disrupted mitochondrial oxidative phosphorylation, contributing to reduced seed-setting rate, decreased plant height, and drought sensitivity observed in the mutants.

The Western blot analysis revealed significant differences in protein levels between the wild-type (WT) and *ppr674* mutants. Using IDH2 as the mitochondrial loading control, the levels of Cytochrome C were markedly reduced in the mutants compared to the WT plants (Figure 5D). This reduction in Cytochrome C indicates a potential impairment in the mitochondrial electron transport chain, specifically in complex III, as Cytochrome C plays a crucial role in shuttling electrons between complex III and complex IV [27]. To confirm the RNA-binding capability of OsPPR674, RNA electrophoretic mobility shift assays (REMSA) were performed using purified *OsPPR674*-GST protein and three FITC-labeled RNA probes targeting the *ccmC*-299 site (Figure 5E–G). The results showed binding of OsPPR674 to the *ccmC* transcript at the site of probe3, confirming its direct involvement in RNA editing.

These findings suggest that the loss of *OsPPR674* leads to disruptions in the normal editing of *ccmC*, thereby affecting the stability and activity of cytochrome c oxidase. This disruption impairs the mitochondrial electron transport chain, particularly complex III, resulting in reduced plant height, lower seed set, and decreased drought tolerance.

## 3. Discussion

In this study, we characterized *OsPPR674*, a mitochondrial-localized PPR protein, and demonstrated its crucial role in rice growth, development, and drought tolerance. Our molecular analysis indicated that *OsPPR674* is crucial for the regulation of RNA editing at the *ccmC*-299 site within the mitochondrial transcript, a modification that is essential for the synthesis of cytochrome c and the proper functioning of mitochondria [28]. The disruption of this editing process in *OsPPR674* knockout plants led to a deficiency in cytochrome c, which in turn impaired mitochondrial respiration and resulted in an accumulation of reactive oxygen species (ROS), ultimately enhancing the plants’ sensitivity to drought stress. Furthermore, under drought conditions, the upregulation of SOD and POD activities in the *OsPPR674* knockout lines signifies the plant’s defensive response to ROS, while the downregulation of proline content suggests impaired osmoregulation. Notably, previous research has established that the restriction of cytochrome oxidase (COX) and alternative oxidase (AOX) pathways within the mitochondrial electron transport chain (ETC) significantly increases the susceptibility to stress in drought-sensitive varieties [29]. Collectively, these findings underscore the pivotal role of *OsPPR674* in modulating drought tolerance in rice, potentially through its regulatory effects on mitochondrial function and energy metabolism. The importance of RNA editing in plant stress tolerance is further highlighted by research on wheat, which revealed that variations in the editing efficiency of the *NAD9* gene influence mitochondrial stability and function, thereby affecting drought tolerance [30]. Additionally, the phenotypic similarities between the *OsPPR674* knockout plants and those with altered expression of *OsBAG6*, a mitochondrial chaperone regulator, or the *PPS1* gene—both of which lead to reduced plant height—emphasize the integral role of mitochondrial function in rice growth and reproduction [31,32]. Our study thus highlights the critical contribution of *OsPPR674* to the preservation of mitochondrial integrity and energy metabolism, which are essential for maintaining optimal plant performance under stress conditions.

The PPR protein family plays a key role in RNA metabolism in plant organelles, with E2-type PPR proteins primarily involved in RNA editing and splicing [33]. To further explore the potential functional redundancy within the PPR E2 family, we conducted RT-qPCR analysis on genes located in the same phylogenetic branch as *OsPPR674* (Figure 1A and Supplementary Figure S4). The results revealed a significant upregulation in the expression of four genes in the *ppr674* mutant, suggesting the potential activation of a compensatory mechanism. Among these, we focused on *PPR756*, the closest homolog of *OsPPR674* based on evolutionary relationships. Expression patterns revealed that *PPR756* is mainly expressed in reproductive organs, while *OsPPR674* shows lower expression across various tissues (Figure 1B). This suggests that *PPR756* is crucial during reproduction, as evidenced by pollen sterility and reduced seed setting in *PPR756* knockout plants due to impaired RNA editing at mitochondrial genes *atp6*, *ccmC*, and *nad7* [25]. In contrast, *OsPPR674* knockout plants had lower seed-setting rates, shorter plant heights, and increased drought sensitivity. The *OsPPR674* mutants failed to exhibit a phenotype analogous to that of *ppr756* mutants, presumably attributable to divergent expression patterns. RT-qPCR analysis showed high expression of O*sPPR674* in roots (Figure 2A), which are vital for water and nutrient uptake and interact with soil microorganisms. Mitochondrial function in roots is essential for supporting growth by driving energy metabolism and mitigating abiotic stresses, such as drought and salinity. Through aerobic respiration, mitochondria generate ATP, which fuels root metabolism and helps maintain cellular redox balance by regulating reactive oxygen species (ROS) levels [34,35]. Moreover, the differences between *OsPPR674* and *PPR756* phenotypes may stem from *OsPPR674* editing only one of *PPR756’s* three target genes at a different site. Notably, the *EMP601* protein in maize influences seed development by editing the *ccmC* gene, essential for mitochondrial function and seed maturation [26]. Research also indicates a negative correlation between the efficiency of RNA editing and a plant’s drought tolerance, with significant alterations in the editing of the *ccmC* gene observed following drought treatment [24]. These findings elucidate the indispensable role and functional versatility of PPR proteins in mitochondrial RNA editing, which is fundamental to sustaining mitochondrial integrity and, in turn, crucial for normal plant growth and the plant’s ability to cope with abiotic stresses. Moreover, even a single mitochondrial gene can exhibit diverse phenotypic effects due to variations in RNA editing at specific sites and the associated editing efficiencies.

While this study focused on drought tolerance, *OsPPR674* likely has broader roles in mediating plant responses to other abiotic stresses, such as salinity and cold. Emerging evidence highlights that RNA editing in plant organelles is highly responsive to environmental changes, with changes in RNA editing efficiency linked to stress acclimation [5,36]. The compensatory upregulation of related PPR proteins observed in our study suggests that functional redundancy may serve as a broader strategy to maintain mitochondrial function under stress. Disruption of RNA-editing processes has been shown to impair mitochondrial function and thereby reduce plant fitness under stress conditions [30]. Our findings demonstrate that the loss of RNA editing at the *ccmC*-299 site in the *ppr674* mutant underscores the critical role of this editing event in mitochondrial function and drought stress tolerance. Such findings are consistent with previous studies in rice, demonstrating that mutations in RNA-editing-associated proteins reduce stress resistance.

Future investigations should explore *OsPPR674*’s role in salinity and cold stress tolerance through transcriptome-wide RNA-editing analysis and mutant screens. Additionally, exploring the interaction network of *OsPPR674* with other PPR proteins and stress-signaling pathways may uncover novel regulatory mechanisms induced by abiotic stress. Furthermore, systematic profiling of RNA-editing events under various stress conditions could elucidate how RNA editing enhances organelle adaptability, ultimately improving plant stress tolerance.

## 4. Materials and Methods

### 4.1. Plant Materials and Growth Conditions

The Japonica rice cultivar 9522 was used for transgenic analysis and was preserved by the laboratory. The *Osppr674* mutants were generated in 9522 (wild type) using a CRISPR/Cas9 system. Based on the coding sequence of *OsPPR674* (LOC_Os08g03676), the guide RNA (gRNAs) targets in *OsPPR674* and primers were selected via the E-CRISP Design Tool (http://www.e-crisp.org) accessed on 10 January 2021. Two single-guide RNAs (sgRNAs)—sgRNA-1 (5′-GTCGTCGAAGACGAGGCGCGCGG-3′) and sgRNA-2 (5′-GATGCTGCATGAGGCGGAGTTGG-3′)—were chosen for gene editing and incorporated into the CRISPR/Cas9 vector pYLCRISPR/Cas9Pubi-H. The sequence of the resulting vector was verified and introduced into *Agrobacterium tumefaciens* EHA105 to infect the 9522. The transgenic rice lines were subsequently confirmed by sequencing the PCR products, and two mutant lines, *ppr674#1* and *ppr674#2,* were used for subsequent phenotypic assays. The wild-type and transgenic plants were grown under natural field conditions from July to November 2024 in Changsha, China (28°11′ N, 112°58′ E). The number of branches per panicle and plant height were measured from 10 plants in the paddy fields at full maturity. The spike length and seed-setting percentage were measured manually. The samples’ normal distribution was assessed using GraphPad Prism 9.0 (San Diego, CA, USA) software via a Shapiro–Wilk test. Data were statistically analyzed via one-way ANOVA followed by Dunnett’s multiple comparisons test with adjusted p values or student’s *t*-test using GraphPad Prism 9.0 software.

### 4.2. Phenotypic Analysis of Mutants

Phenotypic observations and measurements of the *ppr674* mutants were conducted, including key agronomic traits such as seed-setting rate, panicle length, number of primary branches per panicle, and plant height. Precision tools, such as calipers and measuring tapes, were used for data collection and compared against 9522 (wild type). To assess drought tolerance, 14-day-old seedlings were utilized to simulate water-deficit conditions by applying a 20% PEG6000 solution.

### 4.3. Phylogenetic Analysis of the Rice PPR E2 Family

The rice PPR E2 family has been previously reported by Chen et al. (2018) [8]. For phylogenetic analysis, the protein sequences of 69 E2 subfamily genes from the rice PPR family were aligned using MEGA11 (Tempe, AZ, USA), and a phylogenetic tree was constructed using the Maximum Likelihood method. Based on their characteristics, these genes can be divided into three distinct clades [37].

### 4.4. Gene Expression Analysis

Expression patterns of *OsPPR674* and its homologous gene *PPR756* were analyzed using bioinformatics methods. Expression data were obtained from the Rice Expression Database (https://ngdc.cncb.ac.cn/red/, accessed on 9 February 2025), which provides gene expression profiles under various tissues, developmental stages, and environmental conditions [38]. After downloading, the data underwent preprocessing to eliminate background noise and were normalized to ensure accuracy. Subsequently, TBtools-II (College of Horticulture, South China Agricultural University, Guangzhou, China) was utilized to plot the expression profiles, which were then polished with Adobe Illustrator (AI) 2025 (Adobe Inc., San Jose, CA, USA) for enhanced clarity and visual appeal [39]. Expression levels across various tissues were compared to elucidate the potential functions of *OsPPR674*.

RT-qPCR was conducted to verify the expression of *OsPPR674* in various tissues, specifically the root, stem, and leaves, as well as at different stages of panicle development (P4–P7). Total RNA was extracted and reverse-transcribed using the HiScript II Q Select RT SuperMix for qPCR (+gDNA wiper) (Vazyme, Nanjing, China). RT-qPCR was conducted on a LightCycler 480 System (Roche, Basel, Switzerland) using the SYBR^®^ Premix Ex Taq™ II (Tli RNaseH Plus) kit (Takara, Kusatsu, Japan), and relative expression levels were analyzed.

### 4.5. Subcellular Localization Analysis

To determine the subcellular localization of the OsPPR674 protein, the *OsPPR674* gene was fused with GFP and inserted into an expression vector [40]. The 35S::*OsPPR674*-GFP construct was introduced into rice protoplasts using PEG-mediated transformation. After overnight incubation at 28 °C, green fluorescence signals were observed using a Zeiss LSM 880 inverted confocal microscope (Carl Zeiss AG, Oberkochen, Germany). Mitochondrial markers, including MitoTracker Orange and *COX11*-mCherry, were used for co-localization experiments to confirm the subcellular localization of OsPPR674.

### 4.6. Physiological Parameter Measurements

Physiological parameters, including proline content, superoxide dismutase (SOD) activity, peroxidase (POD) activity, and malondialdehyde (MDA) content, were measured in *ppr674* mutants and WT plants under normal and drought conditions. Appropriate kits and protocols were used to evaluate physiological responses and antioxidant capacity under abiotic stress.

### 4.7. Mitochondrial RNA-Editing Analysis

RNA-editing analysis was conducted by extracting total RNA from leaves using Trizol reagent, followed by reverse transcription. Primers covering 81 mitochondrial genes were designed, and RT-PCR products were directly sequenced (Supplementary Table S1). Each analysis was repeated in triplicate.

### 4.8. RNA Electrophoretic Mobility Shift Assay (REMSA)

The cDNA fragment of *OsPPR674* was amplified using primers listed in the Supplementary Table S1 and cloned into the pGEX-4T-1 vector to generate recombinant GST-*PPR674*. RNA probes were synthesized and FITC-labeled at the 3′ end by yKang (Hangzhou, China). RNA-binding assays were performed using the Biotime RNA EMSA Kit (GS606) (Beyotime, Shanghai, China) to assess the interaction between the recombinant protein and RNA probes [41].

### 4.9. Immunoblotting Analysis

Total protein was extracted from rice leaves using RIPA lysis buffer and separated by SDS-PAGE [42]. Proteins were transferred to PVDF membranes (MilliporeSigma, Billerica, MA, USA) and incubated with primary antibodies, including CytC, IDH2 (Abconal, Beijing, China). Signals were detected using ECL Western Blotting Detection Reagents (Bio-Rad, Hercules, CA, USA).

## 5. Conclusions

In conclusion, this study highlights the critical role of *OsPPR674* in ensuring proper mitochondrial function through its involvement in the RNA editing of key mitochondrial transcripts. Disruption of *OsPPR674* expression has an adverse impact on plant growth and stress tolerance, underscoring the importance of precise RNA editing in plant growth and adaptation to environmental challenges. Interestingly, our findings also reveal that *PPR756*, which shares the same target gene *ccmC* as *OsPPR674*, exhibits high expression in reproductive organs, while *OsPPR674* is expressed at lower levels in most tissues, with higher expression in mature roots. This suggests that the functional differences between *OsPPR674* and *PPR756* are likely attributed to their distinct expression patterns, which in turn affect the RNA-editing efficiency and mitochondrial function, ultimately leading to different phenotypic outcomes. These findings provide valuable insights into the molecular mechanisms underlying the functional diversity of PPR proteins and their impact on plant growth and stress responses.

## Data Availability

Data is contained within the article and Supplementary Materials.

## Supplementary Materials

The following supporting information can be downloaded at https://www.mdpi.com/article/10.3390/ijms26062646/s1.
