# Supplementary material for: The Pentatricopeptide Repeat Protein OsPPR674 Regulates Rice Growth and Drought Sensitivity by Modulating RNA Editing of the Mitochondrial Transcript ccmC
Source: Int J Mol Sci. 2025 Mar 14;26(6):2646. doi: 10.3390/ijms26062646 (PMC11941812; doi:10.3390/ijms26062646)
Supplement: Supplementary file 1 [file ijms-26-02646-s001.zip › OsPPR674_ Supplementary Materials Figures.pdf]

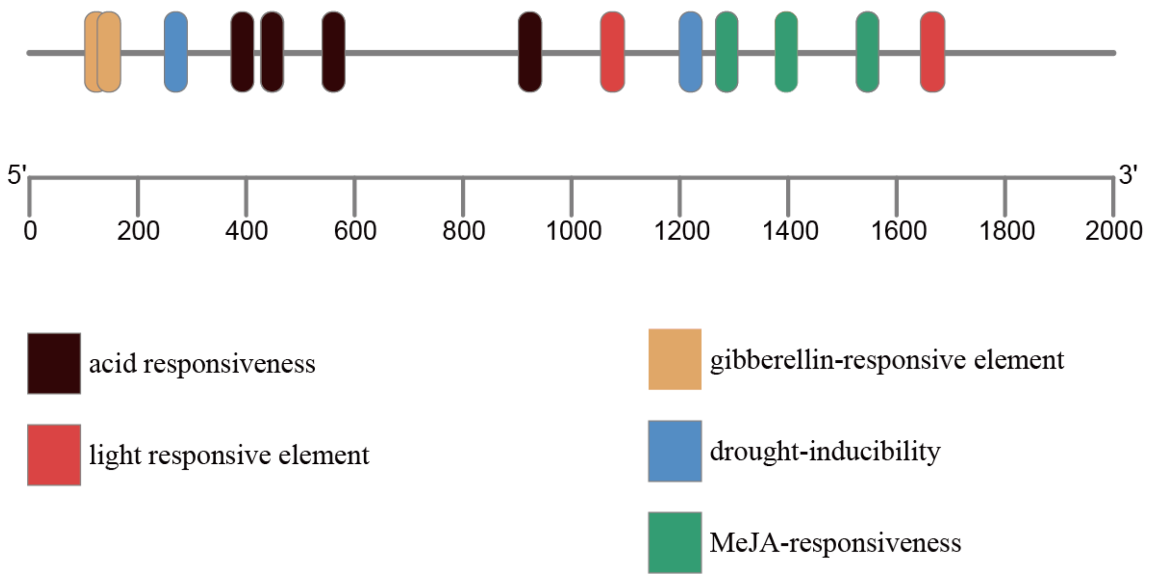

Figure S1. Promoter analysis of *OsPPR674*.
The positions of various response elements in the OsPPR674 promoter are indicated, including acid-responsive, light-responsive, gibberellin-responsive, drought-induced, and methyl jasmonate-responsive elements.

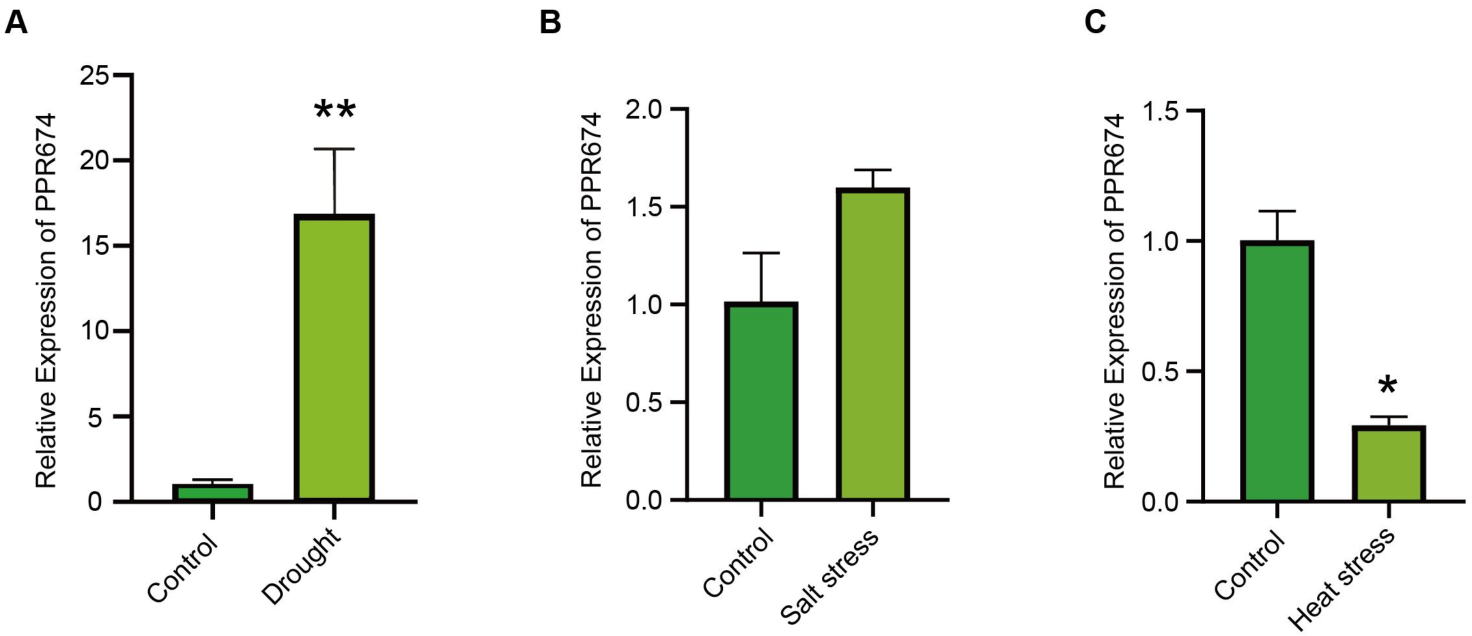

Figure S2. Relative expression of the *PPR674* gene under drought, salt, and heat stress conditions.
(A) Expression levels of the *PPR674* gene in the wild-type strain 9522 after 24 hours of drought treatment (20% PEG6000).
(B) Expression levels of the *PPR674* gene in the wild-type strain 9522 after 24 hours of salt treatment (150 mM NaCl).
(C) Expression levels of the *PPR674* gene in the wild-type strain NIP after 24 hours of heat stress treatment (45°C).
Note: Asterisks indicate statistical significance: *$P < 0.05$, **$P < 0.01$ (Student's t-test).

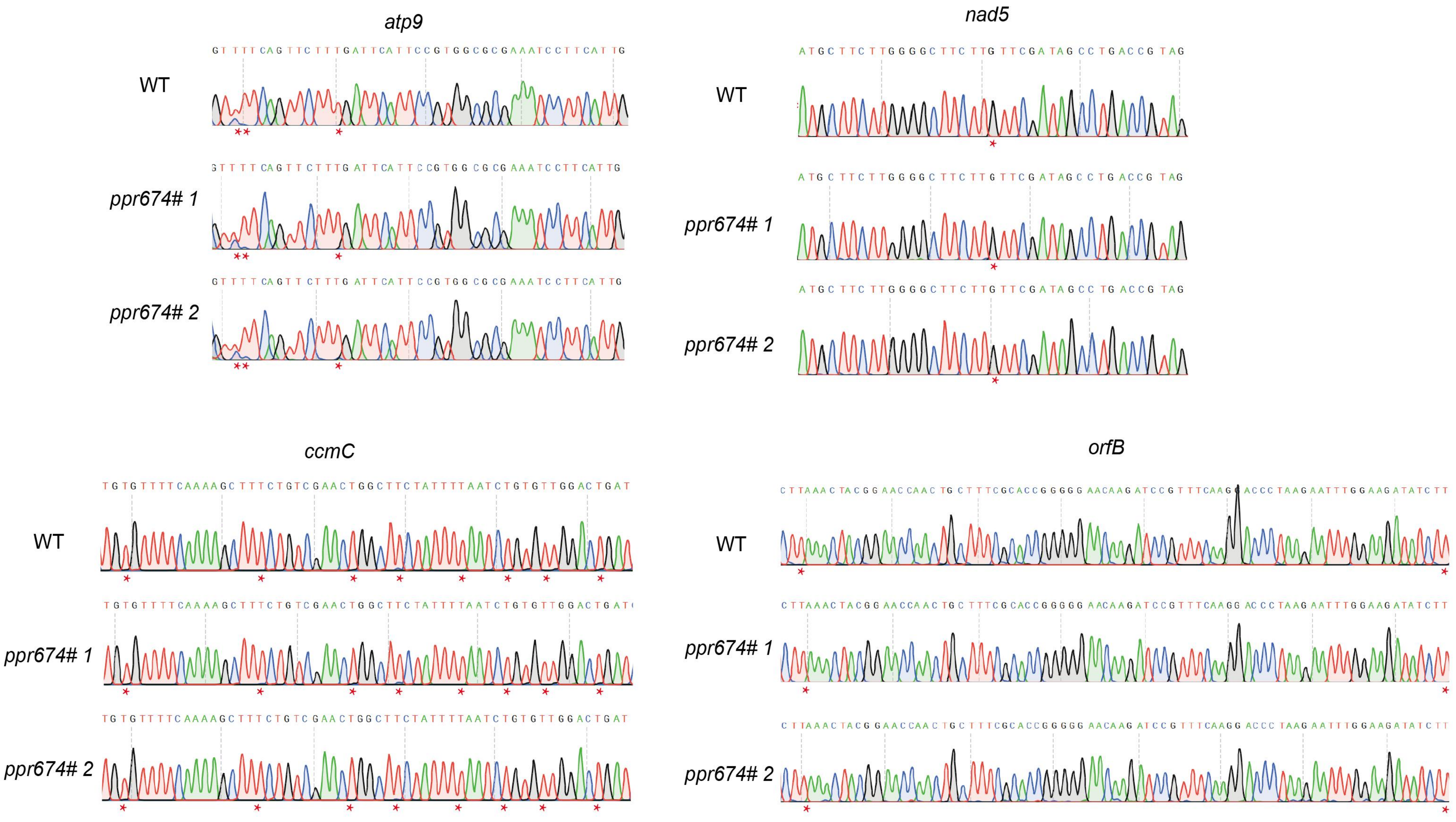

Figure S3. RNA editing analysis of unaffected sites in the WT and ppr674 mutant lines.
RNA editing analysis of unaffected mitochondrial edited sites in the WT and ppr674 mutant line. The edited sites are marked by “ * ”.

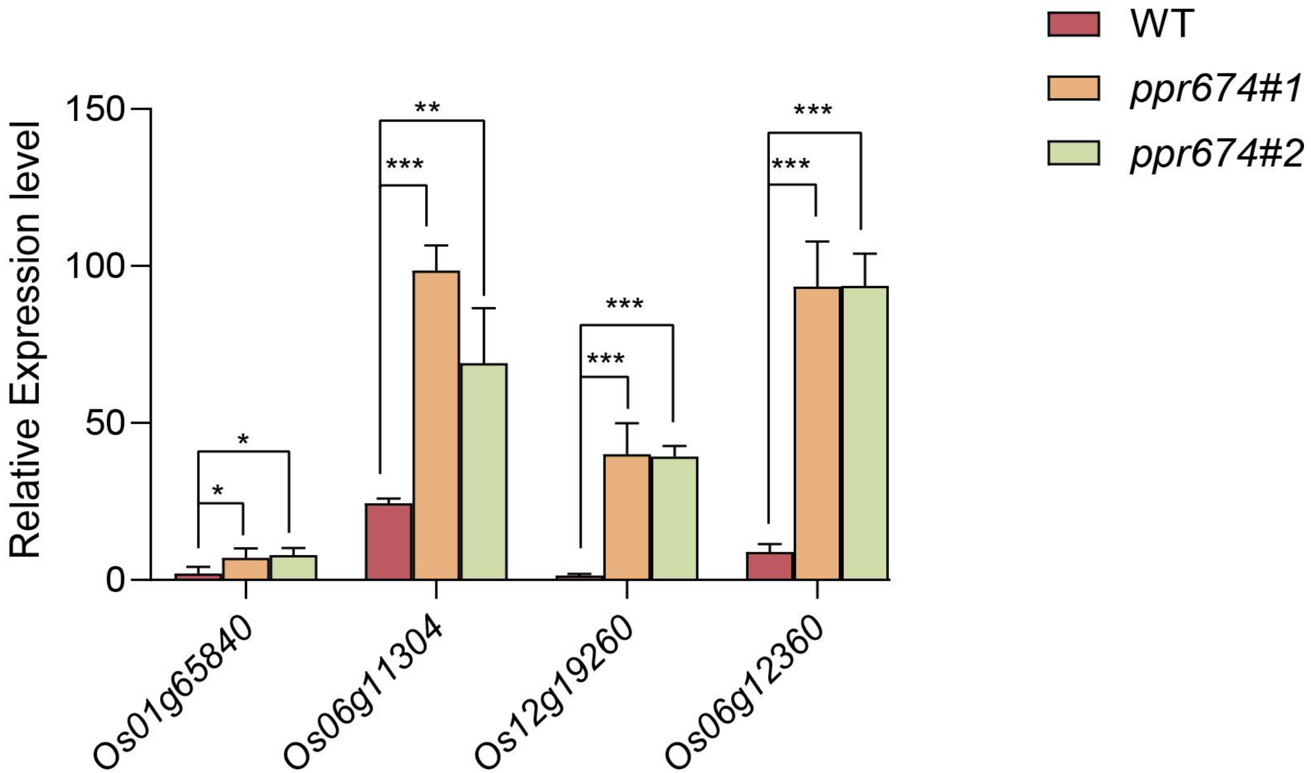

Figure S4. Expression analysis of PPR protein E2 family genes in the same phylogenetic clade as PPR674 in WT and mutant lines. Expression levels of PPR protein family genes in the same clade as PPR674 were compared between WT and PPR674 mutant lines. Note: Asterisks indicate statistical significance: *P < 0.05, **P < 0.01, ***P < 0.001 (ANOVA followed by Tukey's HSD test).
