# Supplementary material for: The Pentatricopeptide Repeat Protein OsPPR674 Regulates Rice Growth and Drought Sensitivity by Modulating RNA Editing of the Mitochondrial Transcript ccmC
Source: Int J Mol Sci. 2025 Mar 14;26(6):2646. doi: 10.3390/ijms26062646 (PMC11941812; doi:10.3390/ijms26062646)
Supplement: Supplementary file 1 [file ijms-26-02646-s001.zip › OsPPR674_Supplementary Materials Table.pdf]

Table S1. Primers used in the *OsPPR674* study.

Primer name	Primer sequences(5'-3')	Application
OsPPR674-F1	CCTCAACGCGCTGCTCAC	Genotype *ppr674#1* mutant
OsPPR674-R1	CCGAGATGATGGCACCGTAG	
OsPPR674-F2	CCTCAACGCGCTGCTCAC	Genotype *ppr674#2* mutant
OsPPR674-R2	CCGAGATGATGGCACCGTAG	
OsPPR674-F3	CCAGTCTTGCCAGAGAAGGG	RT-PCR analysis of *OsPPR674* gene
OsPPR674-R3	CGCCCTTTTTGCATCAACCA	
OsPPR674-F4	GCGCTGCGACTGCCAAGAG	*OsPPR674* cloning into pCAMBIA1300 for subcellular localization
OsPPR674-R4	TTCAGTGGCCTTATCCTGCA	
OsPPR674-F5	CGCTGCGACTGCCAAGAG	*OsPPR674* cloning into Pgex-4T-1 for RNAEMSA
OsPPR674-R5	CTATTCAGTGGCCTTATCCTGCA	
COX11-F1	GTGGAGGAGAAGATCTCACG	*COX11* cloning into pBI121MCS-mCherryy for subcellular localization
COX11-R1	CGTGAGATCTTCTCCTCCAC	
OsActin-F1	AACCAGCTGAGGCCCAAGA	Internal normalization
OsActin-R1	ACGATTGATTTAACCAGTCCATGA	
1-*trnp*-F	TCGAGTCTTTATCGATCGGG	Mitochondrial RNA editing assay
1-*trnp*-R	GGCTACGGGGCTTTGC	
2-*nad1*-F	GGAAATACTTTGTTTAATTCTACC	
2-*nad1*-R	CCAGCAACTGATTCAGCTT	
3-*cox3*-F	ATACAACCGGGGCAAAGT	
3-*cox3*-R	ACCTCCCCACCAATAGATAGA	
4-*orf25*-F	TTGAGTTCAACGGATAAGAAG	
4-*orf25*-R	CTTAGGACTATCAAGCCTTCC	
5-*orf152a*-F	GTCCGAAGAACACTTGCCC	
5-*orf152a*-R	AAACCAATTGAAATGGAAGAGTAG	
6-*nad5*-F	CTCGGTAGTTCCGTAGCC	
6-*nad5*-R	AAGAAAACTGCTCACTAACAAA	
7-*trnV*-F	TAACCTTTGATTAGTTCCTTG	
7-*trnV*-R	TCTTTTCGTTCTATGAACTTTA	
8-*trnM*-F	TCTGACTTGACTCAGCTTGA	
8-*trnM*-R	TCTGTGCTTGCTGTCATG	
9-*trnH*-F	GAGAAAATCCTTTAGCTGGATAAG	
9-*trnH*-R	AACTAGGAATATGGAAAATTGCA	
10-*trnP*-F	ACACTGTACAACAATTTGGAAA	
10-*trnP*-R	CCCTCGTTACTGCCCAT	
11-*trnW*-F	AATCATCCTTTTCTTTACTGC	
11-*trnW*-R	TTTTGATTCGACATAACAAGA	
12-*trnQ*-F	TTGCTTCTCCCTTCCCC	

12-*trnQ*-R	TTTATTAGGGATGAGACTTTCTTA	
13-*rps7*-F	GGGACTTTGATGGTGAGC	
13-*rps7*-R	TCCGATTGGTGGAAGC	
14-*orf490*-F	ATGGATGAAGAGTATGGTGCTT	
14-*orf490*-R	TTCTAGGTCGGTTACAGGTGAA	
15-*orf258*-F	AGCCGTTAGGGAGAATGACTGA	
15-*orf258*-R	CAAAGCAGGTCGAGCCCAC	
16-*orfB*-F	ATGCCTCAACTTGATAAATT	
16-*orfB*-R	AGATTATGCTTCCTTGCC	
17-*trnM*-F	CAGTCTGTGCTTGCTGTCA	
17-*trnM*-R	TCTGACTTGACTCAGCTTGT	
18-*orf181*-F	CTGCTGATAAAGATGGACGAA	
18-*orf181*-R	GCCAACCGCACATGAATT	
19-*nad6*-F	TGTCTAGCCCTGCTTTGG	
19-*nad6*-R	AGATTTGGTCTGTCGTCCTC	
20-*trnI*-F	AAAACCTAGTTCACTCGCTAA	
20-*trnI*-R	TTGGTGGGCGCTTTC	
21-*ccmC*-F	CAGTTTCGTTATTACAACCTTAT	
21-*ccmC*-R	TTCCTTCTCGAGCTTCTATT	
22-*orf183*-F	TTTGATAAAAGTATAGAGCAGGAA	
22-*orf183*-R	GCTTTGGATCATCGCTAAGT	
23-*trnI*-F	CTTTCTGTCCGCTCTTCCC	
23-*trnI*-R	GAGTGGTTCAGGTGACTACCG	
24-*trnD*-F	ACTGTTGAATCGACAGTAGTTACG AA	
24-*trnD*-R	CAGCTTCCAGTGAAGACAAAATG	
25-*trnN*-F	TGGTCCAATGGCTAAAGCTC	
25-*trnN*-R	ACCAAGGACTGAGTTCCCACT	
26-*trnK*-F	TGAATCTGCTTCGGTATCTC	
26-*trnK*-R	GGCTTCGCCCTTCAATA	
27-*orfX*-F	TACTCATTGAATGGAATTTCGC	
27-*orfX*-R	TTCTAATCGTCCAGCCCTC	
28-*nad7*-F	AACGGGCAAATCAAGAAT	
28-*nad7*-R	TCTTGAGTACCTATGATGGTGAC	
29-*orf288*-F	ATCCGATAATCATTCTACTTATTCTT CT	
29-*orf288*-R	ATCCTCATCCGCCCGTAA	
30-*orf194*-F	TTTGAAAGAGGCAGACCCC	
30-*orf194*-R	GATCCTTCCAAGGCACAGG	
31-*rps3*-F	TCAGATCCAAGTCGGTTCAGT	
31-*rps3*-R	GGATACTTCCGCAAGAGCA	
32-*rpl16*-F	ATGCTCTTGCGGAAGTATCCA	

32-*rpl16*-R	GCTAATCTAGCGGCTTGTCG	
33-*nad3*-F	GCGAGAAACGGCTATGAAA	
33-*nad3*-R	CTCCCGATCCGAAGCAC	
34-*rps12*-F	AATTGATTCGTCATGGTAGAG	
34-*rps12*-R	TTTCGATTTTGGTCTTTCTG	
35-*orf224*-F	TTCTCACAATTCTTCTGGTTCTG	
35-*orf224*-R	TCAAGTTTCAAGTCTGCTTCTTATA	
36-*rps2*-F	AAAAGACCAATCAAATCAAACT	
36-*rps2*-R	ATAAGATCCTGAAAACTAAGGG	
37-*trnfM*-F	GGGAAAGAAAGACCAGGGG	
37-*trnfM*-R	GGTGATCAGGACTCGTAGAAGTAT	
38-*nad4*-F	GAATGCTATTTCGATCTAAGTG	
38-*nad4*-R	ATTTGCCATGTTGCACTAA	
39-*cox2*-F	ATTCCTCACAATCGCTCTTT	
39-*cox2*-R	ACCCAATCCGCATAATCT	
40-*orf161*-F	CATGCTACGGCGGATAA	
40-*orf161*-R	TCCAGGAAATTATGAAATGAA	
41-*orf152b*-F	CCTTCCCTCCAAGCACATA	
41-*orf152b*-R	TGAAGCGTTCCTACCATCC	
42-*orf187*-F	ATGACCCGCATCCTACAAC	
42-*orf187*-R	GGACTCGAAATCCTCTTTAGCT	
43-*atp6*-F	ATGAATTTCGATCACAATCA	
43-*atp6*-R	ATACAAATTGAGATCGTAGAAAC	
44-*orf153*-F	ATGTCTAGGGCTTCATCTTATG	
44-*orf153*-R	TCGGGTTCTTATTATTATGTTCT	
45-*rps13*-F	GAGCTAGATCACTTCCCGATGA	
45-*rps13*-R	AGCTTGCGAGCAGTCCTT	
46-*rps4*-F	CGTGTCGTCTACTTCCAGG	
46-*rps4*-R	CGTCCGTTTCCGCTCC	
47-*rps11*-F	CTGATAGGCCTGGGCTTG	
47-*rps11*-R	GGTAGAGGGAGGAGTCGG	
48-*orf176*-F	AAGGCAAGGAGTGTAAACCACG	
48-*orf176*-R	GCTGCAAGAACAGAGGGAGGC	
49-*trnS*-F	CGAGCGCAGGGATGGA	
49-*trnS*-R	ATGACCTCGCGGATGGAG	
50-*atp9*-F	ATGTTAGAAGGAGCTAAATCAATAGG	
50-*atp9*-R	GAAAACGAATGAAATCAGAAAGG	
51-*rrn5*-F	GTGATTGATAAACAAGAAGGAAAATG	
51-*rrn5*-R	TGAAGCCAACATCCAGCCT	
52-*rrn18*-F	AAATCTGAGTTTGATCCTGGCTC	

52-*rrn18*-R	TCCCCTACGGCTACCTTGTT	
53-*rpl2*-F	GCGCTTAGACATTTCACTTTGA	
53-*rpl2*-R	TCCTGCCCGAAATCCTG	
54-*rps19*-F	GATTTCGGGCAGGAGTGG	
54-*rps19*-R	GGGCTGGTCGAGTATGAAGA	
55-*nad4L*-F	ACGGATCCTATAAAATATTT	
55-*nad4L*-R	TTAACCTTGAATGCAATT	
56-*orf241*-F	CTTAATGTTGTGCAGATGGTTG	
56-*orf241*-R	TTGCTGCTCCTCCTCCC	
57-*Cob*-F	CTATAAGGAACCAACGATTCT	
57-*Cob*-R	CCGGTGCGATGAGTCT	
58-*mat-r*-F	CGAAGTTTAGACCGCTCACAGTAG	
58-*mat-r*-R	GTAGTTGAGTGCTCCGTCGTTG	
59-*rps1*-F	ATGTTCTTGGTGGATGCAG	
59-*rps1*-R	GCTATTATCACAATATCCCTCC	
60-*ccmFn*-F	CATTATTTGTTATTTCCGGGTCT	
60-*ccmFn*-R	CAGCCAGTTGCTGGCTC	
61-*ccmFc*-F	ATGGTCCAACTACAGAACTTCTT	
61-*ccmFc*-R	GACGTAACAAACTACGCGAG	
62-*trnS*-F	AACCAACCCGGCACATCC	
62-*trnS*-R	TCGCCCGCCCGTTCTACTT	
63-*orf165*-F	TTATGCTTTGTCTCATTCTATCTC	
63-*orf165*-R	GCTGGTTAGAGCAAAGGACT	
64-*orf284*-F	AATGAGACAAAGCATAAAGGG	
64-*orf284*-R	TCTAATTGGAAGATCAGTAGAGTGT	
65-*cox1*-F	TGGTCCGATGGCTCTTCT	
65-*cox1*-R	AGGCTGGAGGGCTTTGTA	
66-*rps14*-F	ATGTCGGAGAAGCGAAATA	
66-*rps14*-R	TTATGCCCATCAAAGAACC	
67-*rpl5*-F	ATGTTTCCACTCCATTTTCA	
67-*rpl5*-R	AAGCCGCTCCACAGTAGT	
68-*atp1*-F	GCTGCGGAACTCACGACT	
68-*atp1*-R	TCTCCTTCGCAGTTTGTTTTA	
69-*trnS*-F	TACGAAAACTCGCTATTCACTC	
69-*trnS*-R	GATACATTGTGGTCGGTGGT	
70-*trnF*-F	TTTTAGTCCCTTTAATTGAC	
70-*trnF*-R	ATACCTATTCGGTAGATCCT	
71-*nad9*-F	GGATAACCAATCCATTTTCCAA	
71-*nad9*-R	TCCGTCGCTACGCTGTT	
72-*trnY*-F	CTTCTTATTCAGGAACCCTTTGT	

72-*trnY*-R	CCTAGGCTTCCGCCTACTTTC	
73-*nad2*-F	ATCTATGGGTCTACTGGAGC	
73-*nad2*-R	AGAGGGAAATGCACCTAA	
74-*orf173*-F	GCTAAGCGCAGTTCTTTGAA	
74-*orf173*-R	TTAGGTGTAATAGGACTCCCAGTTA	
75-*trnE*-F	TGGTCCCTTTCGTCCAG	
75-*trnE*-R	GATAGCGAGCGATCACTCA	
76-*rrn26*-F	GGCTAGTCTGCGCTCTTTGGA	
76-*rrn26*-R	CGTTTAGTACGAGATCGCTTCACA	
77-*orf162*-F	TTTGTACTATTTATTTCACGGTT	
77-*orf162*-R	ATCGCACCAGGTAGAACA	
78-*trnC*-F	CCGATCTAAAATCGAGAAAA	
78-*trnC*-R	TTGGATCAGTACTATAAGCCTAAG	
79-*ccmB*-F	ATGAGACGACTCTTTCTTGAA	
79-*ccmB*-R	TGTGAACTAATCGAGACCGA	
80-*orf160*-F	GAGTTTACCCGGCACTAGC	
80-*orf160*-R	GAATTTTGTTGATCCCCATT	
81-*trnR*-F	CAACCTGGTAGCGAAGG	
81-*trnR*-R	TTGCGTCCAATAGGATTT	
